# Triumph of Pneumococcal Conjugate Vaccines: Overcoming a Common Foe

**DOI:** 10.1093/infdis/jiaa535

**Published:** 2021-09-30

**Authors:** Gail L Rodgers, Cynthia G Whitney, Keith P Klugman

**Affiliations:** 1 Bill & Melinda Gates Foundation, Seattle, Washington, USA; 2 Rollins School of Public Health and Emory Global Health Institute, Emory University, Atlanta, Georgia, USA

**Keywords:** pneumococcal conjugate vaccine, pneumonia, meningitis

## Abstract

Pneumococcal conjugate vaccine (PCV) has reduced the burden of pneumococcal disease by the near elimination of vaccine serotypes from countries giving a booster dose at >9 months of life. Herd protection, induced by interruption of pneumococcal vaccine type transmission has protected children too young to be immunized, children and adults with underlying risk conditions for invasive pneumococcal disease, and the elderly. PCV has rolled out in most poor countries, but millions of children remain un-immunized especially in middle income countries because of cost constraints. These are being met by considering fewer doses to maintain herd protection, and support for more affordable vaccine from developing country manufacturers. While 3rd generation PCV’s with potential inclusion of 20+ serotypes are close to market in adults, it will be their introduction into childhood immunization and herd protection that is most likely to maximize the public health benefits of these vaccines.

In 1901, William Osler referred to pneumonia as the “captain of the men of death” and believed that most pneumonia was caused by *Streptococcus pneumoniae* (the pneumococcus) [1]. At the time the first pneumococcal conjugate vaccine (PCV) was licensed a century later, the pneumococcus remained the most common cause of severe pneumonia and bacterial meningitis, leading to a global estimate at that time of 735 000 deaths among children <5 years of age per year [2]. In addition, the emergence of strains resistant to multiple antibiotics was making pneumococcal infections more difficult to treat. The availability of PCV programs revolutionized our ability to prevent pneumococcal disease not only in infants but in entire populations—going well beyond protecting those who directly receive them. In this manuscript, we describe the triumph of PCVs over the former “captain” of serious disease and death, including a look forward at sustaining and building on the success achieved to date.

## PNEUMOCOCCI, PNEUMOCOCCAL DISEASE, AND DISEASE EPIDEMIOLOGY

*Streptococcus pneumoniae* is a Gram-positive diplococcus that is part of the normal flora of the nasopharynx in humans. Pneumococcal colonization is usually asymptomatic, and transmission more often occurs from persons who are colonized than from those with disease. Infants start acquiring pneumococci in the first weeks to months of life, and duration of carriage with a particular strain can range from weeks to months [3]. In low-income settings, pneumococcal colonization can be detected within the first month of life. People who develop pneumococcal disease generally do so after new acquisition of a new pneumococcal strain [4].

Most pneumococcal strains, including all those that cause meningitis and other severe infections, are surrounded by a polysaccharide capsule. Anticapsular antibodies can protect against specific pneumococcal serotypes, and vaccines licensed to date have been made from capsular polysaccharides. The capsule is the bacterium’s most important virulence factor, enabling it to bind to cells and evade the immune system. The hundredth pneumococcal serotype has recently been described [5] based on differences in the polysaccharide structure; the pneumococcal serotypes differ in their ability to cause disease and in how frequently they are identified in the nasopharynx [6]. Pneumococcal carriage is more frequent in children, with lower carriage rates in adults [7]; thus, children are the main reservoir and transmitters of pneumococci [8]. Serotypes that are often found in pneumococcal carriage studies of young children are more often resistant to antibiotics than other serotypes [9]. A significant proportion of pneumococci show some reduced susceptibility to penicillin, although many of the strains with intermediate penicillin resistance remain susceptible to expanded-spectrum cephalosporins; full resistance to macrolides and trimethoprim-sulfamethoxazole is more common.

Pneumococci cause disease when they spread or invade beyond their natural reservoir in the nasopharynx. Otitis media and sinusitis result from local spread of pneumococci within the upper respiratory tract; these syndromes are the most common pneumococcal infections and, conversely, pneumococci are the most common bacterial cause of these syndromes. Otitis media is the most common reason for antimicrobial treatment among children in the United States (US) [10]; although most episodes of otitis media are mild, recurrent episodes can lead to hearing loss. More serious disease syndromes like meningitis, bacteremia, and sepsis result from invasion of pneumococci through the nasopharynx or other upper airway structures; infections of these and other normally sterile body sites are collectively referred to as “invasive disease” or invasive pneumococcal disease (IPD). Pneumonia can result from aspiration of pneumococci or spread from the upper to lower airway, often in conjunction with other bacteria and viruses. Pneumococcal meningitis can have case fatality rates of 25% or more in some settings and can lead to long-term disabilities such as blindness and hearing loss, but more pneumococcal deaths and hospitalizations occur as a result of pneumonia, as it is a much more common presentation of pneumococcal disease [11].

**Figure 1.**
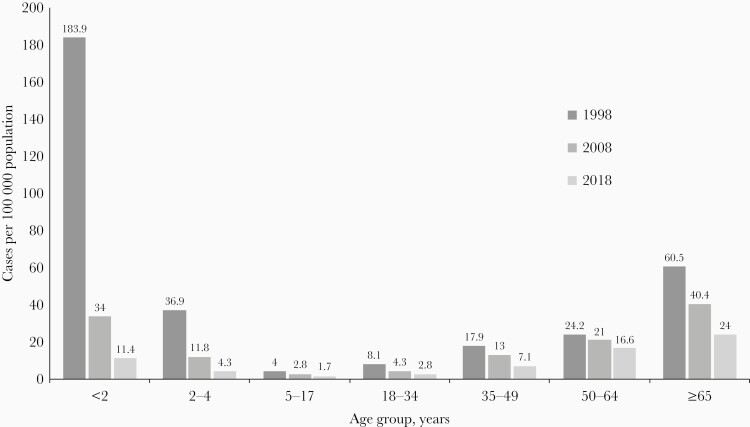
Incidence of invasive pneumococcal disease in the United States (US) by age group and year, from the Centers for Disease Control and Prevention’s Active Bacterial Core surveillance. The first-generation pneumococcal conjugate vaccine (7-valent PCV) became part of the routine US infant immunization program in 2000 and was replaced with a second-generation vaccine (13-valent PCV) in 2010.

The epidemiology of pneumococcal disease shows a U-shaped distribution for disease risk by age, with IPD rates more common in young children and the elderly than in older children and young or middle-aged adults [12] (Figure 1) Pneumococcal disease, particularly pneumococcal pneumonia, continues to be an important cause of morbidity and mortality, especially in these high-risk age groups. Recent global data have emphasized the overall decrease in deaths caused by lower respiratory infections since PCVs and *Haemophilus influenzae* type b (Hib) vaccine became available, but this decrease has been seen more so among children than among adults. For those >70 years of age, mortality remains high, with an estimated 1 129 400 deaths due to lower respiratory infections in 2017, a 33.6% increase from 2007. Modeled trends in deaths due to pneumococcal pneumonia show large differences between children and adults; comparing 1990 to 2017, there was a 71.2% decrease in children aged <5 years and a 60.4% increase in those >70 years of age. Therefore, the pneumococcus remains an important cause of morbidity and mortality as the high-risk adult population over age 65 increases globally [13].

While anyone can get pneumococcal disease, those with immunocompromising conditions or certain chronic illnesses are at higher risk than healthy persons of similar age. Those with untreated human immunodeficiency virus (HIV) or leukemia have disease rates as much as 50–100 times higher than immunocompetent persons, and smoking or conditions such as diabetes and kidney failure increase risk 2- to 5-fold [14]. Those with sickle cell disease and asplenia have increased disease rates and higher risk of severe sepsis if infected. In the US, persons of black race or native Americans have disease rates 2–4 times those of white persons [15].

Pneumococcal disease risk varies by season. In temperate climates, pneumococcal disease rates peak in colder months in temperate zones and in dry seasons more so than in rainy seasons in other climates. Pneumococcal disease rates vary geographically as well, with higher rates of pneumonia and meningitis, in particular, in sub-Saharan African countries than in North America or Europe [16]. Nearly all deaths from pneumonia in children occur in sub-Saharan Africa and South Asia. In countries within the African meningitis belt, pneumococcal meningitis outbreaks have been reported, in particular outbreaks caused by serotype 1. Although a pneumococcal meningitis outbreak was reported in Ghana in recent years, such outbreaks are uncommon now that PCV is included in the national immunization programs in most African countries [17].

Serotypes commonly causing disease can differ somewhat by geography as well, with serotypes 1 and 5 occurring more often in low- and middle-income populations than in high-income populations. A global review of serotypes causing IPD in children <5 years of age found that the amount of disease caused by serotypes targeted by the first-generation 7-valent conjugate vaccine (containing serotypes 4, 6B, 9V, 14, 18C, 19F, and 23F) ranged between 50% and 80% by region; adding serotypes 1 and 5 and other serotypes in the 10- and 13-valent second-generation conjugates increased coverage to 60%–90% in all regions [18]. The diversity of serotypes causing disease is greater among adults than among children.

## THE ROAD TO CONJUGATE VACCINES

Early versions of pneumococcal vaccines were made from purified polysaccharide capsules, and the literature shows some benefit controlling pneumococcal outbreaks among miners in South Africa [19] and military recruits during World War II in the US [20]. The first pneumococcal vaccine recommended for use among adults in the US was made from polysaccharides of 14 pneumococcal serotypes. In 1983, the recommendation was updated to include vaccines made from 23 serotypes, a product still in use for high-risk groups among persons 2 years and older. Pneumococcal vaccines of this design were never recommended for children <2 years of age, however, because polysaccharide antigens do not induce a robust immune response in infants and toddlers.

A vaccine made from attaching polysaccharide from Hib to a carrier protein was introduced to the market in 1987. This vaccine was highly effective at preventing Hib meningitis, even in infants, and provided the design inspiration for future PCVs. In contrast to the T-cell–independent, mostly B-cell immune response induced by pure polysaccharide vaccines, attaching polysaccharide to a carrier protein creates vaccines that induce T-cell–dependent immune responses, which are typically robust even in young infants and can be boosted with subsequent doses [21].

The first commercially available PCV was a 7-valent formulation (PCV7; Wyeth Lederle Vaccines) that included serotypes most often identified as causes of IPD in the US (4, 6B, 9V, 14, 18C, 19F, 23F). A phase 3 clinical trial that included nearly 38 000 infants in the US showed high efficacy (97%) for a 4-dose schedule against IPD caused by vaccine serotypes in addition to serotype 6A, with significant efficacy against pneumonia and otitis media [22, 23]. A related PCV that also included serotypes 1 and 5 (PCV9; Wyeth Lederle Vaccines) was tested in clinical trials in The Gambia and South Africa, and again showed efficacy against IPD, pneumonia, and vaccine-type carriage [24, 25, 26]; of note is that PCV9 prevented 16% of deaths from any cause among the vaccinated group in the Gambian trial. Additionally, the South African trial demonstrated that children who had received PCV9 were significantly less likely to be hospitalized for pneumonia in which influenza or other viruses were detected, demonstrating how pneumococcal vaccines can prevention hospitalizations for viral-pneumococcal coinfections [27]. A smaller, cluster-randomized trial among Navajo and Apache children in the US noted efficacy against IPD but did not demonstrate protection against pneumonia, a divergent finding that has never been fully explained [28]. Another first-generation conjugate that included 11 serotypes was tested in the Philippines, but the low efficacy against pneumonia in a phase 3 trial led to the withdrawal of the product from further development [29]; interestingly, the efficacy of the product was higher for those who had to travel farther to receive treatment [30]. Results from a clinical trial in the US evaluating maternal immunization with PCV9 and infant immunization with PCV7 found a higher risk for otitis media among infants <6 months of age whose mothers received PCV9 during pregnancy, suggesting that maternal antibody blunts the infant PCV7 immune response [31].

The World Health Organization (WHO) established criteria for licensing of PCVs for use in children based on a primary endpoint of immunoglobulin G (IgG) antibody concentration and secondary endpoints of functional activity measured by opsonophagocytic activity and induction of immunologic memory [32]. Because the first 3 clinical trials of the Wyeth-Lederle products (PCV7 in Northern California Kaiser Permanente and American Indians and PCV9 in South Africa) also included assessments of antibody response, researchers were able to determine what amount of antibody was associated with protection against disease—a so-called correlate of protection. Pooled results of the >66 000 subjects in these 3 studies demonstrated an efficacy of 93% for IPD and determined that an IgG antibody level of 0.35 µg/mL measured 4 weeks after the third primary series dose correlated with protection [33, 34]. Since then, the US Food and Drug Administration and other licensing bodies have used that value to license new products without requiring demonstration of efficacy against IPD, although the requirements for demonstration of comparable immunogenicity also require demonstration of functional antibody activity using an opsonophagocytic assay.

After PCV7 was licensed, demand increased for second-generation PCVs covering more serotypes, in particular serotypes 1 and 5, types that cause a high burden of illness and death in low-income settings. GlaxoSmithKline marketed a product (PCV10-GSK) containing 10 serotypes (PCV7 serotypes plus 1, 5, and 7F). Bridging studies of antibody levels showed comparability of PCV10-GSK to PCV7, and clinical trials in Finland (The Finnish Invasive Pneumococcal disease [FinIP] trial) and Latin American countries (Clinical Otitis Media & Pneumonia Study [COMPAS] trial) demonstrated the efficacy of PCV10-GSK against IPD, pneumonia, carriage, and otitis media; vaccination also reduced the need for antibiotics in young children [35, 36]. Pfizer, which had acquired Wyeth Lederle, the original maker of PCV7, licensed a 13-valent vaccine (PCV13) that included the 10-valent serotypes along with serotypes 3, 6A, and 19A. Demand for PCV13 was driven in part by an antibiotic-resistant 19A strain that emerged in the US after PCV7 introduction and was reported in several other countries [37]. PCV13 was licensed for infants using the correlate of protection principle and comparison of immune responses to those of PCV7 [38].

Surveillance data from countries implementing national PCV immunization programs demonstrated that such programs prevented disease not only in those receiving vaccination, but also among unvaccinated children and adults in the populations through herd (indirect) protection (discussed further below). Although PCVs were originally designed for pediatric use, clinical trials in adults have shown their ability to prevent disease in persons of all ages, via direct protection. In Malawi, PCV7 was shown to prevent IPD in a clinical trial among adults with advanced HIV disease and recurrent pneumococcal IPD [39]. PCV13 was licensed for adults based on a clinical development program in the US and Europe consisting of 6 studies that evaluated PCV13 compared to 23-valent polysaccharide vaccine (PPSV23) among adults 50 years of age and older, evaluating those who were naive to pneumococcal vaccine and those who had received 1 or more doses of PPSV23. These trials involved healthy subjects and those with chronic conditions. Results demonstrated noninferior or statistically superior opsonophagocytic activity, the standard for protection in adults, to PPSV23 for serotypes in common as well as demonstration of a T-cell response by induction of immune memory that enhances subsequent vaccination with either PCV13 or PPSV23 [38, 40]. A subsequent clinical trial, the Community-Acquired Pneumonia Immunization Trial in Adults (CAPITA) trial, included >80 000 adults aged 65 years and older in the Netherlands and demonstrated efficacy against IPD (75%) and pneumonia (45%) caused by vaccine serotypes [41].

Following the CAPITA trial, the US implemented a national recommendation for routine use of PCV13 in series with PPSV23 among adults aged 65 years and older in 2014, 4 years after PCV13 introduction for infants [42]. Surveillance data from the US found that, in the setting of an established routine infant immunization program with the same vaccine, no further reduction in PCV13 vaccine-type IPD occurred following the introduction of PCV13 for adults, primarily because few vaccine-type strains remained in circulation [13]. Less clear is the PCV13 serotype-specific nonbacteremic pneumonia burden, although recent studies suggest that 3.3% of cases in those >65 years of age were due to vaccine serotypes, which may translate to a large number of cases [43], Overall, only minimal changes in the incidence of pneumococcal disease among adults were seen at the population level following routine use of PCV13 in adults. In light of these data, the US recommendation for PCV13 in all older adults without additional risk factors was made optional for physicians under a category of shared clinical decision making [44]. Under these guidelines, adults who have potentially increased risk of exposure to PCV13 serotypes, such as those residing in long-term health facilities, residing in areas with low pediatric PCV13 uptake, or traveling to settings with no pediatric PCV program; those with chronic heart, lung, or liver disease; those with diabetes or alcoholism; and those who smoke cigarettes or who have >1 chronic medical condition should consider PCV13 vaccination in conjunction with their physician [44].

## GLOBAL USE OF PCVS AND THEIR EFFECTIVENESS

Populations that introduced PCVs in infants noted rapid and dramatic decreases in IPD and pneumonia, as well as in otitis media. Decreases in IPD in children following introduction of PCV7 in the US were evident within 6 months of its introduction and were also demonstrated in other countries using PCV7. Effectiveness was also documented for high-risk groups such as those with sickle cell disease [45]. Importantly, PCV introduction significantly reduced the racial disparity in disease incidence between white persons and individuals of black race [46] and indigenous populations [47].

A decrease in vaccine-type carriage with subsequent decreases in transmission and disease among unimmunized persons, known as herd effects, was anticipated following introduction of PCV7 because herd effects had been seen earlier with Hib vaccination, and reduction of vaccine-type carriage had been demonstrated following infant immunization with PCV9 [26]. The first hint that PCV7 rolled out at a national scale in infants could decrease disease in unimmunized persons came from the Centers for Disease Control and Prevention’s Active Bacterial Core surveillance in about 2003, with later publications showing the full extent of herd effects [48]. Analyses of large databases of pneumonia hospitalizations in the US found that a reduction in pneumonia hospitalization had also occurred among adults, in addition to decreases in IPD [49, 50]. Modeled data based on US administrative data estimated a reduction of nearly 800 000 hospitalizations for pneumococcal pneumonia, with 90% of these occurring through herd effects among adults; specifically, 64% of the reduction was in those aged 65 and older [49]. Thus, remarkably, the largest public health impact of PCV7 in reducing hospitalization and mortality in that study in the US was in the elderly, perhaps not surprisingly, given the large burden of adult pneumonia among older adults.

While the impact of PCV7 in countries that introduced it was remarkable, the global public health benefits of PCV7 were limited due in part to supply constraints and policy makers waiting for formulations that included serotypes 1 and 5, types prevalent in geographies with large disease burdens. In addition, populations that had introduced PCV7 had noted an increase in colonization and, to a lesser extent, disease caused by pneumococcal serotypes that the vaccines do not target—a concept known as “serotype replacement.” Following PCV7 introduction, serotype replacement occurred in both adults and children, particularly due to serotype 19A [37].

**Figure 2.**
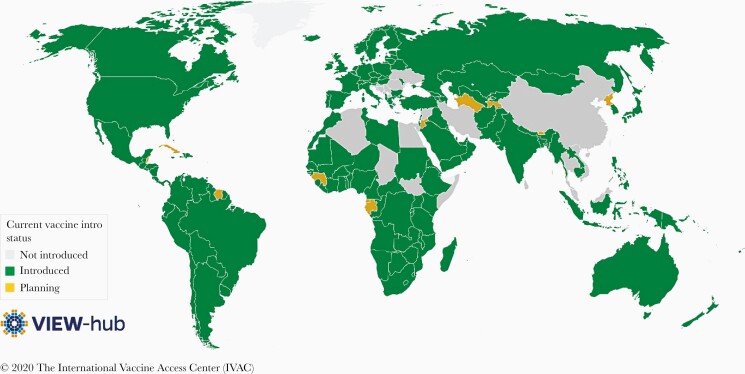
Current pneumococcal conjugate vaccine introduction status, by country. Accessed at www.view-hub.org, 1 July 2020.

PCV10-GSK and PCV13 rapidly replaced PCV7 in countries that had existing PCV programs as soon as these formulations were licensed in 2009 and early 2010. In addition, many countries adopted these second-generation PCVs after they were recommended by the WHO for inclusion into national immunization programs globally. Currently, PCVs have been incorporated into the national immunization programs of 145 countries [51] (Figure 2). PCV13 has been incorporated into 77% of national programs, PCV10-GSK in 19%, and both in 4%. Due to the innovative funding mechanism of Gavi’s Advanced Market Commitment, PCV10-GSK and PCV13 were introduced in low-income (Gavi) countries at the same time as in high-income countries, avoiding the usual 10- to 15-year delay in low-income countries where the burden of disease is greatest; currently, 92% of high-income countries and 82% of Gavi countries have PCV programs [52]. The high price of PCVs for middle-income countries, which cannot access Gavi prices, has led to introduction in only 66% of middle-income countries [52]. Affordability is the biggest current obstacle in those countries yet to introduce PCVs, although other factors exist such as concern that introduction of another vaccine may overburden a fragile Expanded Programme on Immunization (EPI) program and/or may not be acceptable due to multiple injections. Lower-cost alternatives for PCVs remain an important option for upper-middle–income countries as well as those lower-middle–income countries graduating from Gavi support. Several countries have expressed interest in changing to a novel, recently WHO-prequalified, less expensive 10-valent PCV that includes serotype 19A, produced by the Serum Institute of India (Table 1).

**Table 1.** Approved Pneumococcal Conjugate Vaccines (PCVs) and Extended-Valency PCVs Currently in Human Clinical Trials

Vaccine	Status	Serotype																							
		1	3	4	5	6A	6B	7F	9V	14	18C	19A	19F	23F	22F	33F	8	9N	10A	11A	12F	15B	2	17F	20B
PCV10 (GSK)	Approved	X	…	X	X	…	X	X	X	X	X	…	X	X	…	…	…	…	…	…	…	…	…	…	…
PCV10 (Serum Institute of India)	Approved	X	…	…	X	X	X	X	X	X	…	X	X	X	…	…	…	…	…	…	…	…	…	…	…
PCV13 (Pfizer)	Approved	X	X	X	X	X	X	X	X	X	X	X	X	X	…	…	…	…	…	…	…	…	…	…	…
PCV15 (GSK)	Phase 3	X	X	X	X	X	X	X	X	X	X	X	X	X	X	X	…	…	…	…	…	…	…	…	…
PCV 20 (Pfizer)	Phase 3	X	X	X	X	X	X	X	X	X	X	X	X	X	X	X	X		X	X	X	X	…	…	…
PCV24 (Affinivax)	Phase 1/2	X	X	X	X	X	X	X	X	X	X	X	X	X	X	X	X	X	X	X	X	X	X	X	X

Abbreviations: PCV, pneumococcal conjugate vaccine.

The global introduction of second-generation PCVs has afforded the opportunity to demonstrate their effectiveness globally. Both vaccines have demonstrated effectiveness against IPD, pneumonia, and otitis media as well as in high-risk populations and have shown evidence of herd effect [53, 54]. The impact of PCVs in high-burden countries have been transformative and have mirrored the effects seen in high-income countries for IPD and pneumonia, the largest killer of children worldwide. As an example, following routine introduction of PCV13 in The Gambia, chest radiograph–confirmed pneumonia dropped by 24%, hypoxic pneumonia by 61%, and pneumococcal pneumonia by 86% [55].

Impact of the PCV on mortality has been documented in high-income countries such as the US [56], but effectiveness against mortality from pneumonia in high-mortality settings has been only inferred from the original PCV9 trial in The Gambia, which showed a 16% reduction in deaths from all causes [24]. Recently, across Latin America where a reasonable amount of data is collected on vital statistics, an impact of these 2 vaccines on childhood mortality has been recently documented, ranging from 11% to 35% reductions in deaths from pneumonia in 5 countries [57].

 Although the majority of data come from high-income countries, herd effects seen by PCV10-GSK and PCV13 have been demonstrated in all geographies [53, 54, 58, 59]. A systematic review found that higher baseline rates of disease and higher PCV coverage rates in children were associated with higher levels of herd effect and, as noted with PCV7, the main beneficiaries of indirect protection are those aged >65 years [60].

Similarly, while PCV9 demonstrated for the first time an impact on antibiotic-resistant IPD [25] and PCV10-GSK similarly has shown effectiveness against antibiotic-resistant IPD in Finland [61], PCV10-GSK was also shown in a large cluster randomized trial in Finland to reduce antibiotic use in children [62]. A recent analysis of antibiotic consumption in households in low- and middle-income countries that have PCV programs found a 19.7% reduction in antibiotic use for respiratory infection [63].

## CONTINUED CHALLENGES

Despite their high degree of success, work is ongoing to optimize use of PCVs and minimize serotype replacement.

### Schedule Optimization

Evaluation of the PCV schedules, both in moving to booster-containing schedules and reducing doses, is an area of intense investigation. The pivotal PCV7 efficacy study was conducted using a schedule of 3 primary doses with a booster (3 + 1), and this schedule was adopted by the US and a small number of other high-income countries initially [22]. Shortly after PCV7 introduction in the US, however, an extended shortage resulted in many infants receiving fewer than the recommended 3 doses; a case-control study conducted during this time demonstrated effectiveness against IPD of 1 and 2 doses given at age <7 months of 73% and 96%, respectively, compared to no vaccination, although protection from a single early dose was not sustained after 12 months of age [64]. To accommodate the EPI schedule in many European countries, a study of PCV7 administered in a reduced schedule of 2 primary doses with a booster (2 + 1) in the United Kingdom (UK) demonstrated comparable immunogenicity to the 3 + 1 schedule following the booster dose [65]; these immunogenicity data, along with the successful experience with a 2 + 1 schedule from Quebec [66], prompted many countries to adopt this schedule. Two efficacy studies of an investigational PCV9 conducted in The Gambia [24] and South Africa [25] used the dosing schedule common to most Gavi countries, 3 doses without a booster (3 + 0), and demonstrated efficacy against IPD and pneumonia. Therefore, currently, these 3 dosing schedules—3 + 1, 2 + 1, and 3 + 0—are approved and used globally and have all demonstrated effectiveness for prevention of pneumococcal disease. Although all schedules have been shown to decrease vaccine-type carriage leading to herd effects, the magnitude of these effects differs by schedule. In high-income countries using booster-containing schedules, either 3 + 1 or 2 + 1, there has been a nearly complete elimination of circulation of vaccine serotypes [67, 68], whereas the low-income countries using a primary 3 + 0 dose schedule only, show continued high residual carriage of vaccine serotypes despite good vaccine coverage [69]. Booster dosing may be particularly important in prevention of disease due to certain serotypes such as serotype 1, which greatly decreased in both vaccinated and unvaccinated populations in South Africa with a 2 + 1 regimen [58] but continued as an important cause of disease in Ghana, a country with very good PCV coverage of a 3 + 0 schedule [70]. Currently, several countries in Africa have elected to change from 3 + 0 to a 2 + 1 schedule, and continued surveillance of disease and carriage will provide important data regarding the ability of booster doses to better control pneumococcal disease through herd protection in these low-income settings.

Data suggest that use of catch-up campaigns in children can accelerate the impact of PCVs since reductions in vaccine type carriage in 3- to 5-year-olds may be the best predictor of impact in IPD [8]. This was evident in Kilifi, Kenya, where introduction of PCV10 with a catch-up program in 1- to 5-year-olds showed subsequent residual vaccine type carriage of 6%, lower than residual vaccine-type carriage found in other countries using a 3 + 0 schedule [59]. Given the anticipated effects of vaccinating toddlers, Cuba is planning to introduce its indigenous PCV7 by immunizing preschool-aged children only. The effect of the program on infants, through herd protection, remains to be evaluated.

Recently, there has been interest in reducing the number of doses in the infant PCV schedule for countries that have adequate control of disease and low vaccine-type carriage. The main drivers of this change are cost reduction as well as the need for space for additional vaccines in a crowded immunization schedule. A reduced schedule of 2 doses (1 + 1) could potentially maintain low circulation of vaccine serotypes and continued disease control. The 1 + 1 schedule demonstrated comparable immunogenicity to a 2 + 1 schedule in the UK [71] and in South Africa [72]. In January 2020, the UK changed its infant immunization schedule to 1 + 1, and the effectiveness of this approach will be assessed through its national surveillance system. The Bill & Melinda Gates Foundation is funding additional studies to evaluate the immunogenicity and effect on carriage of a 2-dose regimen (1 + 1) in The Gambia, India, and Vietnam. Further data on vaccine-type disease and carriage are needed to elucidate the cost–benefit criteria for a 2-dose schedule and whether this schedule can be a more affordable, booster-containing regimen for maintenance of pneumococcal vaccine-type herd protection in low-income countries.

### Serotype Replacement

Data from the UK, which switched from PCV7 to PCV13, demonstrated a reduction in burden of IPD due to vaccine types in both children and in adults, through herd protection, although the reduction in adult disease due to vaccine serotypes was less than that observed in children [73]. In addition, the amount of remaining disease caused by PCV13 serotypes has plateaued at a relatively higher level than the reduction seen post-PCV7, when vaccine-type IPD was reduced in children by >97%. The reason for this difference in impact is that PCV13 is poorly effective, at best, against serotype 3 and its ability to reduce serotype 19A through herd protection, while present, has been slow [73]. A similar analysis published in 2016 in the US has shown that among all ages, 5 years after the rollout of PCV13, residual PCV13 serotypes (plus cross-protection from serotype 6A in the vaccine to serotype 6C, not in the vaccine) were still 28.2% of invasive isolates, consisting almost entirely of serotype 3, with smaller amounts of 19A and 19F [74]. Although some degree of cross-protection for PCV10-GSK against serotype 19A has been demonstrated, herd effects have not been seen and surveillance in countries using it have shown increases in serotype 19A disease [75].

Among remaining nonvaccine-type pneumococcal disease, 2 multiresistant serotypes are prominent in the US and elsewhere and are a cause for concern, given that they are not included in the PPSV23 or any currently licensed pneumococcal vaccine, and also not included in a number of putative next-generation vaccines. These are serotype 35B [76], which first emerged as a major problem in children in the US, and serotype 24F, which emerged as a major problem among children with meningitis in France [77]. Serotype 2, named as such as it was the second most common serotype causing IPD in the pre–antibiotic era, and a serotype not recorded in the US for decades, has emerged as a cause of IPD in geographies as disparate as Bangladesh, Guatemala, and Israel [78–80]. Two other replacement serotypes beyond 24F among children and adults in the UK are serotypes 8 and 12F [81]. In Japan, among children, similarly, serotype 24F is now the most important serotype causing IPD plus serotypes 12F, 15A, and 15 B/C [82]. All of these emerging serotypes will need consideration for inclusion in a third-generation pneumococcal conjugate vaccine [83].

## THE FUTURE

To address the need of expanded serotype coverage and serotype replacement, a non-serotype-specific approach, such as a protein-based or whole cell vaccine, would be ideal. Thus far, clinical efficacy studies of protein-based vaccines have failed to protect against otitis media [84] and nasopharyngeal carriage [85]. Therefore, most vaccines currently in late-stage development are serotype-based conjugates. Third-generation PCVs ranging from 15- to 24-valent formulations are in human clinical trials (Table 1); additionally, 25- and 30-valent vaccines are in preclinical development. Plans suggests that 15- and 20-valent conjugate vaccines in development will be submitted initially for licensure for use in adults. If licensed, policy makers will need to weigh the benefits of direct protection among adults for the new serotypes over that already provided indirectly by infant PCV programs. In addition, policy discussions will need to address whether conjugate vaccines covering this number of serotypes can be used instead of PPSV23 to protect older adults and adults with high-risk conditions.

## CONCLUSIONS

In the 20 years since the first PCV came to market, these vaccines have shown their remarkable ability to protect the infants who receive them and, by interrupting transmission of vaccine-type pneumococci in communities, protect persons of all ages in every corner of the world. The rapid uptake of these vaccines into routine infant immunization programs in both wealthy and in low-income settings is a notable achievement that has come about through global cooperation of public health leaders, vaccine manufacturers, governments, and funders. PCVs have, indeed, contributed to removal of the title of “captain of the men of death” from pneumonia and the pneumococcus. Looking forward, however, more can be done to reduce pneumococcal morbidity and mortality. The anticipated availability in the next few years of more PCV products at affordable prices should encourage the remaining countries who have not yet established routine PCV programs to start them. In addition, next-generation formulations may address remaining pneumococcal disease in settings with effective programs.

## Supplementary Data

Supplementary materials are available at *The Journal of Infectious Diseases* online. Consisting of data provided by the authors to benefit the reader, the posted materials are not copyedited and are the sole responsibility of the authors, so questions or comments should be addressed to the corresponding author.

The complete references are available as online Supplemental Material.

## Supplementary Material

jiaa535_suppl_Supplementary-Material

## Notes

***Supplement sponsorship.*** This supplement is sponsored by the Bill and Melinda Gates Foundation.

***Potential conflicts of interest.*** All authors: No reported conflicts of interest.

## References

[19] Austrian R, Douglas RM, Schiffman G, et al. Prevention of pneumococcal pneumonia by vaccination. Trans Assoc Am Physicians 1976; 89:184–94. 14433

[22] Black S, Shinefield H, Fireman B, et al. Efficacy, safety and immunogenicity of heptavalent pneumococcal conjugate vaccine in children. Northern California Kaiser Permanente vaccine study center group. Pediatr Infect Dis J 2000; 19:187–95. 10749457 10.1097/00006454-200003000-00003

[24] Cutts FT, Zaman SM, Enwere G, et al; Gambian Pneumococcal Vaccine Trial Group. Efficacy of nine-valent pneumococcal conjugate vaccine against pneumonia and invasive pneumococcal disease in The Gambia: randomised, double-blind, placebo-controlled trial. Lancet 2005; 365:1139–46. 15794968 10.1016/S0140-6736(05)71876-6

[25] Klugman KP, Madhi SA, Huebner RE, Kohberger R, Mbelle N, Pierce N; Vaccine Trialists Group. A trial of a 9-valent pneumococcal conjugate vaccine in children with and those without HIV infection. N Engl J Med 2003; 349:1341–8. 14523142 10.1056/NEJMoa035060

[27] Madhi SA, Klugman KP; Vaccine Trialist Group. A role for *Streptococcus pneumoniae* in virus-associated pneumonia. Nat Med 2004; 10:811–3. 15247911 10.1038/nm1077 PMC7095883

[35] Palmu AA, Jokinen J, Borys D, et al. Effectiveness of the ten-valent pneumococcal *Haemophilus influenzae* protein D conjugate vaccine (PHiD-CV10) against invasive pneumococcal disease: a cluster randomised trial. Lancet 2013; 381:214–22. 23158882 10.1016/S0140-6736(12)61854-6

[36] Tregnaghi MW, Sáez-Llorens X, López P, et al; COMPAS Group. Efficacy of pneumococcal nontypable *Haemophilus influenzae* protein D conjugate vaccine (PHiD-CV) in young Latin American children: a double-blind randomized controlled trial. PLoS Med 2014; 11:e1001657. 24892763 10.1371/journal.pmed.1001657 PMC4043495

[41] Bonten MJ, Huijts SM, Bolkenbaas M, et al. Polysaccharide conjugate vaccine against pneumococcal pneumonia in adults. N Engl J Med 2015; 372:1114–25. 25785969 10.1056/NEJMoa1408544

[48] Whitney CG, Farley MM, Hadler J, et al; Active Bacterial Core Surveillance of the Emerging Infections Program Network. Decline in invasive pneumococcal disease after the introduction of protein-polysaccharide conjugate vaccine. N Engl J Med 2003; 348:1737–46. 12724479 10.1056/NEJMoa022823

[49] Simonsen L, Taylor RJ, Young-Xu Y, et al. Impact of pneumococcal conjugate vaccination of infants on pneumonia and influenza hospitalization and mortality in all age groups in the United States. MBio 2010; 2:e309–10. 10.1128/mBio.00309-10 PMC3025524 21264063

[50] Griffin MR, Zhu Y, Moore MR, Whitney CG, Grijalva CG. U.S. hospitalizations for pneumonia after a decade of pneumococcal vaccination. N Engl J Med 2013; 369:155–63. 23841730 10.1056/NEJMoa1209165 PMC4877190

[58] von Gottberg A, de Gouveia L, Tempia S, et al; GERMS-SA Investigators. Effects of vaccination on invasive pneumococcal disease in South Africa. N Engl J Med 2014; 371:1889–99. 25386897 10.1056/NEJMoa1401914

[59] Hammitt LL, Etyang AO, Morpeth SC, et al. Impact of ten-valent pneumococcal conjugate vaccine on invasive pneumococcal disease and nasopharyngeal carriage in Kenya: a longitudinal surveillance study. Lancet 2019; 393:2146–54. 31000194 10.1016/S0140-6736(18)33005-8 PMC6548991

[62] Palmu AA, Jokinen J, Nieminen H, et al. Effect of pneumococcal *Haemophilus influenzae* protein D conjugate vaccine (PHiD-CV10) on outpatient antimicrobial purchases: a double-blind, cluster randomised phase 3-4 trial. Lancet Infect Dis 2014; 14:205–12. 24287186 10.1016/S1473-3099(13)70338-4

