# Supplementary material for: Triumph of Pneumococcal Conjugate Vaccines: Overcoming a Common Foe
Source: J Infect Dis. 2021 Sep 30;224(Suppl 4):S352–9. doi: 10.1093/infdis/jiaa535 (PMC8482025; doi:10.1093/infdis/jiaa535)
Supplement: jiaa535_suppl_Supplementary-Material [file jiaa535_suppl_supplementary-material.docx]

1. Osler W. Principles and practices of medicine, 4th edition. New York: D. Appleton and Company, 1901. Page 108.
2. World Health Organization, Immunization, Vaccines and Biologicals. Estimated Hib and pneumococcal deaths for children under 5 years of age, 2000. https://www.who.int/immunization/monitoring_surveillance/burden/estimates/Pneumo_hib_2000/en/index2.html. Page last updated 6 March 2014. Accessed June 6, 2020.
3. Weiser JN, Ferreira DM, Paton JC. Streptococcus pneumoniae: transmission, colonization and invasion. Nat Rev Microbiol 2018 ; 16:355-367. doi: 10.1038/s41579-018-0001-8.
4. Simell B, Auranen K, Käyhty H, et al. The fundamental link between pneumococcal carriage and disease. Expert Rev Vaccines 2012 ; 11:841-855. doi:10.1586/erv.12.53
5. Ganaie F, Saad JS, McGee L, et al. A new pneumococcal capsule type, 10D, is the 100th serotype and has a large cps fragment from an oral streptococcus. mBio 2020 ; 11:e00937-20
6. Brueggemann AB, Muroki BM, Kulohoma BW, et al. Population genetic structure of Streptococcus pneumoniae in Kilifi, Kenya, prior to the introduction of pneumococcal conjugate vaccine. PLoS One. 2013 ; 8(11):e81539. doi:10.1371/journal.pone.0081539
7. Le Polain de Waroux O, Flasche S, Prieto-Moreno D, Edmunds WJ et al. Age-Dependent Prevalence of Nasopharyngeal Carriage of Streptococcus pneumoniae before Conjugate Vaccine Introduction: A Prediction Model Based on a Meta-Analysis. PLoS One 2014 ; https://doi.org/10.1371/journal.pone.0086136
8. Weinberger DM, Pitzer VE, Regev-Yochay G, Givon-Lavi N, Dagan R. Association between the decline in pneumococcal disease in unimmunized adults and vaccine-derived protection against colonization in toddlers and preschool-aged children. Am J Epidemiol 2018 ; 188:160-8.
9. Kim L, McGee L, Tomczyk S, Beall B. Biological and epidemiological features of antibiotic-resistant Streptococcus pneumoniae in pre- and post-conjugate vaccine eras: A United States perspective. Clin Microbiol Rev 2016 ; 29:525–52.
10. Hersh AL, Jackson MA, Hicks LA, et al. Principles of judicious antibiotic prescribing for upper respiratory tract infections in pediatrics. Pediatr 2013 ; 132:1146-54.
11. van de Beek D, Brouwer M, Hasbun R, et al. Community-acquired bacterial meningitis. Nat Rev Dis Primers 2, 16074 2016 ; https://doi.org/10.1038/nrdp.2016.74
12. National Center for Immunization and Respiratory Diseases, Division of Bacterial Diseases. Centers for Disease Control and Prevention, Active Bacterial Core surveillance (ABCs): surveillance reports. https://www.cdc.gov/abcs/reports-findings/surv-reports.html. Page last reviewed June 7, 2020. Accessed on June 20, 2020.
13. GBD 2017 Causes of Death Collaborators. Global, regional, and national age-sex-specific mortality for 282 causes of death in 195 countries and territories, 1980–2017: a systematic analysis for the Global Burden of Disease Study 2017. Lancet 2018 ; 392:1736-88.
14. Kyaw MH, Rose CE Jr, Fry AM, et al. The influence of chronic illnesses on the incidence of invasive pneumococcal disease in adults. J Infect Dis 2005 ; 192:377-86.
15. Wortham JM, Zell E, Pondo T, et al. Racial disparities in invasive Streptococcus pneumoniae infections, 1998-2009. Clin Infect Dis 2014 ; 58:1250-7.
16. World Health Organization. Pneumococcal conjugate vaccines in infants and children under 5 years of age: WHO position paper – February 2019. Weekly Epidemiol Rec 2019 ; 94: 85–104.
17. Soeters HM, Kambiré D, Sawadogo G, et al. Evaluation of pneumococcal meningitis clusters in Burkina Faso and implications for potential reactive vaccination. Vaccine 2020 in press; available online at https://doi.org/10.1016/j.vaccine.2020.06.002
18. Johnson HL, Deloria-Knoll M, Levine OS, et al. Systematic evaluation of serotypes causing invasive pneumococcal disease among children under five: The pneumococcal global serotype project. PLoS Med 2010 ; 7: e1000348. https://doi.org/10.1371/journal.pmed.1000348
19. Austrian R, Douglas RM, Schiffman G. Prevention of pneumococcal pneumonia by vaccination. Trans Assoc Am Physicians. 1976 ; 89:184-94.
20. Macleod CM, Hodges RG, Heidelberger M, Bernhard WG. Prevention of pneumococcal pneumonia by immunization with specific capsular polysaccharides. J Exp Med 1945 ; 82: 445-65.
21. Centers for Disease Control and Prevention. Prevention of pneumococcal disease among infants and children — use of 13-valent pneumococcal conjugate vaccine and 23-valent pneumococcal polysaccharide vaccine. Recommendations of the Advisory Committee on Immunization Practices (ACIP). MMWR 2010 ; 59:1-19.
22. Black S, Shinefield H, Fireman B, et al. Efficacy, safety and immunogenicity of heptavalent pneumococcal conjugate vaccine in children. Northern California Kaiser Permanente Vaccine Study Center Group. Pediatr Infect Dis J 2000 ; 19:187-95.
23. Hansen J, Black S, Shinefield H, et al. Effectiveness of heptavalent pneumococcal conjugate vaccine in children younger than 5 years of age for prevention of pneumonia: updated analysis using World Health Organization standardized interpretation of chest radiographs. Pediatr Infect Dis J 2006 ; 25:779-81.
24. Cutts FT, Zaman SM, Enwere G, et al. Efficacy of nine-valent pneumococcal conjugate vaccine against pneumonia and invasive pneumococcal disease in the Gambia: randomised, double-blind, placebo-controlled trial. Lancet 2005 ; 365:1139–46.
25. Klugman KP, Madhi SA, Huebner RE, Kohberger R, Mbelle N, Pierce N. A trial of a 9-valent pneumococcal conjugate vaccine in children with and those without HIV infection. N Engl J Med 2003 ; 349:1341–8.
26. Mbelle N, Huebner RE, Wasas AD, et al. Immunogenicity and impact on nasopharyngeal carriage of a nonavalent pneumococcal conjugate vaccine. J Infect Dis 1999; 180:1171-6.
27. Madhi SA, Klugman KP; Vaccine Trialist Group. A role for Streptococcus pneumoniae in virus-associated pneumonia. Nat Med 2004 ; 10:811-3.
28. O'Brien KL, Moulton LH, Reid R, et al. Efficacy and safety of seven-valent conjugate pneumococcal vaccine in American Indian children: group randomised trial. Lancet 2003 ; 362:355-61.
29. Lucero MG, Nohynek H, Williams G, et al. Efficacy of an 11-valent pneumococcal conjugate vaccine against radiologically confirmed pneumonia among children less than 2 years of age in the Philippines: A randomized, double-blind, placebo-controlled trial. Pediatr Infect Dis J 2009 ; 28:455–62.
30. Root ED, Lucero M, Nohynek H, et al. Distance to health services modifies the effect of an 11-valent pneumococcal vaccine on pneumonia risk among children less than 2 years of age in Bohol, Philippines. International Journal of Epidemiology 2017 ; 46:706–16.
31. Daly KA, Giebink SG, Lindgren BR, et al. Maternal immunization with pneumococcal 9-valent conjugate vaccine and early infant otitis media. Vaccine 2014 ; 32:6948-55.
32. Jodar L, Butler J, Carlone G, Dagan R, Goldblatt D, Kayhty H et al. Serological criteria for evaluation and licensure of new pneumococcal conjugate vaccine formulations for use in infants. Vaccine 2003 ; 21:3265-72.
33. Siber GR, Chang I, Baker S, Fernsten P, O’Brien KL, Santosham M et al. Estimating the protective concentration of anti-pneumococcal capsular polysaccharide antibodies. Vaccine 2007 ; 25;3816-26.
34. World Health Organization Expert Committee on Biological Standardization. Recommendations to assure the quality, safety and efficacy of pneumococcal conjugate vaccines. Replacement of: TRS 927, Annex 2. 2009. Available at https://www.who.int/biologicals/areas/vaccines/pneumo/Pneumo_final_23APRIL_2010.pdf?ua=1. Accessed June 24, 2020.
35. Palmu AA, Jokinen J, Borys D, et al. Effectiveness of the ten-valent pneumococcal Haemophilus influenzae protein D conjugate vaccine (PHiD-CV10) against invasive pneumococcal disease: a cluster randomised trial. Lancet 2013 ; 381: 214-22.
36. Tregnaghi MW, Sáez-Llorens X, López P, et al. Efficacy of pneumococcal nontypable Haemophilus influenzae protein D conjugate vaccine (PHiD-CV) in young Latin American children: a double-blind randomized controlled trial. PLoS Med 2014 ; 11: e1001657
37. Moore MR, Gertz RE, Jr, Woodbury RL, et al. Population snapshot of emergent Streptococcus pneumoniae serotype 19A in the United States, 2005. J Infect Dis 2008 ; 197:1016-27.
38. Food and Drug Administration. Prevnar 13: clinical review of new product license application. Rockville, MD: US Department of Health and Human Services, Food and Drug Administration; 2010. Available at http://www.fda.gov/BiologicsBloodVaccines/Vaccines/ApprovedProducts/ucm201667.htm
39. French N, Gordon SB, Mwalukomo T, et al. A trial of a 7-valent pneumococcal conjugate vaccine in HIV-infected adults. Malawi Med J 2016 ; 28:115-22.
40. Gruber WC. PREVNAR® 13. Pneumococcal 13-valent Conjugate Vaccine (Diphtheria CRM197 Protein) Prevention of Pneumococcal Disease in Adults ≥50 Years of Age Vaccines and Related Biological Products Advisory Committee 16 November 2011. Available at: https://wayback.archive-it.org/7993/20170114033354/http://www.fda.gov/downloads/AdvisoryCommittees/CommitteesMeetingMaterials/BloodVaccinesandOtherBiologics/VaccinesandRelatedBiologicalProductsAdvisoryCommittee/UCM284609.pdf. Accessed Aug 10, 2020.
41. Bonten MJ, Huijts SM, Bolkenbaas M, et al. Polysaccharide conjugate vaccine against pneumococcal pneumonia in adults. N Engl J Med 2015 ; 372:1114–25.
42. Tomczyk S, Bennett NM, Stoecker C, et al. Use of 13-valent pneumococcal conjugate vaccine and 23-valent pneumococcal polysaccharide vaccine among adults aged ≥65 years: Recommendations of the Advisory Committee on Immunization Practices (ACIP) Morb Mortal Wkly Rep 2014 ; 63:822–5.
43. McLaughlin JM, Jiang Q, Isturiz RE, Sings HL, Swedlow DL, Gessner BD et al. Effectiveness of 13-valent pneumococcal conjugate vaccine against hospitalizations for community-acquired pneumonia in older US adults: A test negative design. Clin Infect Dis 2018 ; 67;1498-1506.
44. Matanock, A, Lee G, Gierke R, Kobayashi, M, Leidner A, Pilishvili T. Use of 13-Valent pneumococcal conjugate vaccine and 23-Valent pneumococcal polysaccharide vaccine among adults aged ≥65 years: Updated recommendations of the Advisory Committee on Immunization Practices. MMWR 2019 ; 68:1069–75.
45. Halasa NB, Shankar SM, Talbot TR, et al. Incidence of invasive pneumococcal disease among individuals with sickle cell disease before and after the introduction of the pneumococcal conjugate vaccine. Clin Infect Dis 2007 ; 44:1428-33.
46. Talbot TR, Poehling KA, Hartert TV, et al. Elimination of racial differences in invasive pneumococcal disease in young children after introduction of the conjugate pneumococcal vaccine. Pediatr Infect Dis J. 2004 ; 23:726-31.
47. Weatherholtz R, Millar EV, Moulton LH, et al. Invasive pneumococcal disease a decade after pneumococcal conjugate vaccine use in an American Indian population at high risk for disease. Clin Infect Dis 2010 ; 50:1238-46.
48. Whitney CG, Farley MM, Hadler J, et al. Decline in invasive pneumococcal disease after the introduction of protein-polysaccharide conjugate vaccine. N Engl J Med 2003 ; 348:1737-46.
49. Simonsen L, Taylor RJ, Young-Xu Y, et al. Impact of pneumococcal conjugate vaccination of infants on pneumonia and influenza hospitalization and mortality in all age groups in the United States. MBio 2010 ; 2:e309-e310.
50. Griffin MR, Zhu Y, Moore MR et al. U.S. hospitalizations for pneumonia after a decade of pneumococcal vaccination. N Engl J Med 2013 ; 369:155-163.
51. https://view-hub.org/map/?set=current-vaccine-intro-status&group=vaccine-introduction&category=pcv; Accessed July 3, 2020.
52. Global Vaccine Market Model Demand Module, Linksbridge, SPC.
53. Mrkvan T, Pelton SI, Ruiz-Guiñazú J, Palmu AA, Borys D. Effectiveness and impact of the 10-valent pneumococcal conjugate vaccine, PHiD-CV: review of clinical trials and post-marketing experience. Expert Review of Vaccines 2018 ; 17:797-818.
54. Esposito S, Principi N. Impacts of the 13-valent pneumococcal conjugate vaccine in children. J Immunol Res 2015 ; 2015:591580. doi:10.1155/2015/591580
55. Mackenzie GA, Hill PCV, Sahito SM, et al. Impact of the introduction of pneumococcal conjugate vaccination on pneumonia in The Gambia: population-based surveillance and case-control studies. Lancet Infect Dis 2017 ; 17:965-73.
56. Pilishvili T, Lexau C, Farley MM, et al. Sustained reductions in invasive pneumococcal disease in the era of conjugate vaccine. J Infect Dis 2010 ; 201:32-41.
57. de Oliveira LH, Shioda K, Valenzuela KT, et al. Declines in pneumonia mortality following the introduction of pneumococcal conjugate vaccines in Latin American and Caribbean countries. Clin Infect Dis 2020, ciaa614, https://doi.org/10.1093/cid/ciaa614.

58. von Gottberg A, de Gouveia L, Tempia S, et al; GERMS-SA Investigators. Effects of vaccination on invasive pneumococcal disease in South Africa. N Engl J Med 2014; 371:1889–99.

59. Hammitt LL, Etyang AO, Morpeth SC, et al. Impact of tenvalent pneumococcal conjugate vaccine on invasive pneumococcal disease and nasopharyngeal carriage in Kenya: a longitudinal surveillance study. Lancet 2019; 393:2146–54.

60. Tsaban G, Ben-Shimol S. Indirect (herd) protection, following pneumococcal conjugated vaccines introduction: a systematic review of the literature. Vaccine 2017; 35:2882–91.

61. Sihvonen R, Siira L, Toropainen M, Kuusela P, Pätäri- Sampo A. Streptococcus pneumoniae antimicrobial resistance decreased in the Helsinki Metropolitan Area after routine 10-valent pneumococcal conjugate vaccination of infants in Finland. Eur J Clin Microbiol Infect Dis 2017;

36:2109–16.

62. Palmu AA, Jokinen J, Nieminen H, et al. Effect of pneumococcal Haemophilus influenzae protein D conjugate vaccine (PHiD-CV10) on outpatient antimicrobial purchases: a double-blind, cluster randomised phase 3-4 trial. Lancet Infect Dis 2014; 14:205–12.

63. Lewnard JA, Lo NC, Arinaminpathy N, Frost I, Laxminarayan R. Childhood vaccines and antibiotic use in low- and middle-income countries. Nature 2020; 581:94–9.

64. Whitney CG, Pilishvili T, Farley MM, et al. Effectiveness of seven-valent pneumococcal conjugate vaccine againstinvasive pneumococcal disease: a matched case-control study. Lancet **2006**; 368:1495–502.

65. Goldblatt D, Southern J, Ashton L, et al. Immunogenicity and boosting after a reduced number of doses of a pneumococcal conjugate vaccine in infants and toddlers. Pediatr Infect Dis J **2006**; 25:312–9.

66. De Wals P, Lefebvre B, Deceunicnk G, Longtin J. Incidence of invasive pneumococcal disease before and during an era of use of three different pneumococcal conjugate vaccines in Quebec. Open Forum Infect Dis **2017**; 4:S465.

67. Loughlin AM, Hsu K, Silverio AL, Marchant CD, Pelton SI. Direct and indirect effects of PCV13 on nasopharyngeal carriage of PCV13 unique pneumococcal serotypes in Massachusetts’ children. Pediatr Infect Dis J **2014**; 33:504–10.

68. Southern J, Andrews N, Sandu P, et al. Pneumococcal carriage in children and their household contacts six years after introduction of the 13-valent pneumococcal conjugate vaccine in England. PLoS One **2018**; 13:e0195799.

69. Swarthout TD, Fronterre C, Lourenço J, et al. High residual carriage of vaccine-serotype S*treptococcus pneumoniae* after introduction of pneumococcal conjugate vaccine in Malawi. Nat Commun **2020**; 11:2222.

70. Bozio CH, Abdul-Karim A, Abenyeri J, et al. Continued occurrence of serotype 1 pneumococcal meningitis in two regions located in the meningitis belt in Ghana five years after introduction of 13-valent pneumococcal conjugate vaccine. PLoS One **2018**; 13:e0203205.

71. Goldblatt D, Southern J, Andrews NJ, et al. Pneumococcal conjugate vaccine 13 delivered as one primary and one booster dose (1 + 1) compared with two primary doses and a booster (2 + 1) in UK infants: a multicentre, parallel group randomised controlled trial. Lancet Infect Dis **2018**; 18:171–9.

72. Madhi SA, Mutsaerts EA, Izu A, et al. Immunogenicity of a single-dose compared with a two-dose primary series followed by a booster dose of ten-valent or 13-valent pneumococcal conjugate vaccine in South African children: an open-label, randomised, non-inferiority trial. Lancet Infect Dis **2020**; S1473-3099(20)30289–9.

73. Waight PA, Andrews NJ, Ladhani SN, Sheppard CL, Slack MP, Miller E. Effect of the 13-valent pneumococcal conjugate vaccine on invasive pneumococcal disease in England and Wales 4 years after its introduction: an observational cohort study. Lancet Infect Dis **2015**; 15:535–43.

74. Beall B, Chochua S, Gertz RE Jr, et al. A population-based descriptive atlas of invasive pneumococcal strains recovered within the U.S. during 2015–2016. Front Microbiol **2018**; 19:2670.

75. Isturiz R, Sings HL, Hilton B, Arguedas A, Reinert RR, Jodar L. Streptococcus pneumoniae serotype 19A: worldwide epidemiology. Expert Rev Vaccines 2017; 16:1007–27.

76. Olarte L, Kaplan SL, Barson WJ, et al. Emergence of multidrug-resistant pneumococcal serotype 35B among children in the United States. J Clin Microbiol 2017; 55:724–34.

77. Levy C, Varon E, Ouldali N, Béchet S, Bonacorsi S, Cohen R. Changes in invasive pneumococcal disease spectrum after 13-valent pneumococcal conjugate vaccine implementation. Clin Infect Dis 2020; 70:446–54.

78. Saha S, Al Emran HM, Hossain B, et al. Streptococcus pneumoniae serotype-2 childhood meningitis in

Bangladesh: a newly recognized pneumococcal infection threat. PLoS One 2012; 7:e32134.

79. Gaensbauer JT, Asturias EJ, Soto M, Holt E, Olson D, Halsey NA; Guatemala Pediatric Bacterial Surveillance Working Group. Pediatric invasive pneumococcal disease in Guatemala City: importance of serotype 2. Pediatr Infect Dis J 2016; 35:e139–43.

80. Dagan R, Ben-Shimol S, Benisty R, et al. 1888. A nationwide outbreak of invasive pneumococcal disease (IPD) caused by a novel Streptococcus pneumoniae serotype 2 (SP2) clone in the PCV13 era, in Israel. Open Forum Infect Dis 2019; 6:S54.

81. Kandasamy R, Voysey M, Collins S, et al. Persistent circulation of vaccine serotypes and serotype replacement after 5 years of infant immunization with 13-valent pneumococcal conjugate vaccine in the United Kingdom. J Infect Dis 2020; 221:1361–70.

82. Nakano S, Fujisawa T, Ito Y, et al. Nationwide surveillance of paediatric invasive and non-invasive pneumococcal disease in Japan after the introduction of the 13-valent conjugated vaccine, 2015–2017. Vaccine 2020; 38:1818–24.

83. Klugman KP, Rodgers GL. A 3rd generation pediatric pneumococcal conjugate vaccine. Lancet Infect Dis 2021; 21: 14-16.

84. Hammitt LL, Campbell JC, Borys D, et al. Efficacy, safety and immunogenicity of a pneumococcal protein-based vaccine co-administered with 13-valent pneumococcal conjugate vaccine against acute otitis media in young children: a phase IIb randomized study. Vaccine 2019; 37:7482–92.

85. Odutola A, Ota MOC, Antonio M, et al. Efficacy of a novel, protein-based pneumococcal vaccine against nasopharyngeal carriage of Streptococcus pneumoniae in infants: a phase 2, randomized, controlled, observer-blind study. Vaccine 2017; 35:2531–42.
